# Activation of the PI3K-AKT Pathway by Old World Alphaviruses

**DOI:** 10.3390/cells9040970

**Published:** 2020-04-15

**Authors:** Eline Van Huizen, Gerald M. McInerney

**Affiliations:** Department of Microbiology, Tumor and Cell Biology, Karolinska Institutet, 17177 Stockholm, Sweden

**Keywords:** metabolism, apoptosis, autophagy

## Abstract

Alphaviruses can infect a broad range of vertebrate hosts, including birds, horses, primates, and humans, in which infection can lead to rash, fever, encephalitis, and arthralgia or arthritis. They are most often transmitted by mosquitoes in which they establish persistent, asymptomatic infections. Currently, there are no vaccines or antiviral therapies for any alphavirus. Several Old World alphaviruses, including Semliki Forest virus, Ross River virus and chikungunya virus, activate or hyperactivate the phosphatidylinositol-3-kinase (PI3K)-AKT pathway in vertebrate cells, potentially influencing many cellular processes, including survival, proliferation, metabolism and autophagy. Inhibition of PI3K or AKT inhibits replication of several alphaviruses either in vitro or in vivo, indicating the importance for viral replication. In this review, we discuss what is known about the mechanism(s) of activation of the pathway during infection and describe those effects of PI3K-AKT activation which could be of advantage to the alphaviruses. Such knowledge may be useful for the identification and development of therapies.

## 1. Introduction

### 1.1. PI3K-AKT Pathway

Viruses activate metabolic pathways in order to meet their needs for production of appropriate macromolecules. Once such pathway is the multifunctional phosphatidylinositol 3-kinases (PI3K)-AKT pathway. Due to its central importance in metabolism but also other cellular functions, this pathway is a common target for viruses [1,2,3,4]. In this review, we describe the activation of the PI3K-AKT pathway by alphaviruses and the consequent cellular effects.

**Figure 1:**
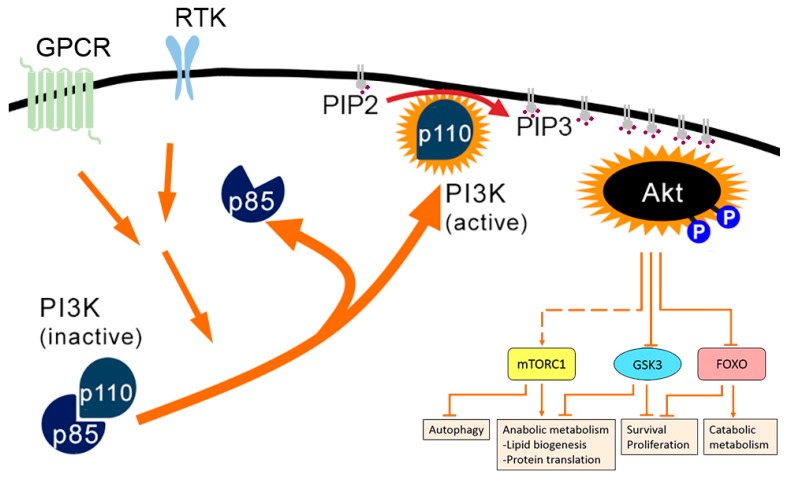
Activation of PI3K-AKT pathway by external signals via G-protein coupled receptors (GPCR) or receptor tyrosine kinases (RTK) leads to dissociation of the p85 regulatory subunit from the active p110 PI3K subunit. The active subunit catalyses the conversion of PIP2 to PIP3 at the plasma membrane, leading to the recruitment and activation of the AKT kinase. Via multiple downstream effector pathways, cellular states of growth, proliferation, heightened metabolic activity and survival are promoted. For more details, see text.

PI3Ks are a large family of kinases that is divided in three classes: class 1 (1A and 1B), class II, and class III. The PI3K-AKT pathway involves class 1A PI3Ks. These PI3Ks are heterodimers with a catalytic subunit (p110) and a regulatory subunit (p85). Class 1A PI3Ks are activated by receptor tyrosine kinases (RTK) or G protein-coupled receptors (GPCR) after binding of growth factors, either directly or indirectly via activation of the small GTPase RAS [5] (Figure 1). Once the regulatory action of p85 has been relieved, active class 1A PI3Ks phosphorylate phosphatidylinositol-4,5-biphosphate (PIP2, or PtdIns(4,5)P_2_) to phosphatidylinositol-3,4,5-triphosphate (PIP3, or PtdIns(3,4,5)P_3_) at the plasma membrane (PM). PIP3-enriched membranes are a docking site for the PI3K-dependent kinase-1 (PDK1) and mTORC2 (or PDK2). Furthermore, the serine/threonine kinase AKT (or protein kinase B, PKB) is relocated to membrane sites with PIP3, where it can be activated via phosphorylation by PDK1 and mTORC2 [6,7]. A number of downstream targets of AKT have been identified, several of which are multifunctional nodes, integrating AKT signalling with signalling through other pathways [7]. One of these is the serine/threonine kinase mammalian target of rapamycin (mTOR) in the multi-protein complex mTORC1. AKT signalling leads to the activation of mTORC1, which promotes cell growth by inducing lipid biogenesis through activation of the transcription factors SREBP1 and PPARγ and by promoting protein synthesis by activating the S6 kinase (S6K) and by inactivating the translational inhibitor 4E-BP1. mTORC1 also inhibits autophagy by blocking ULK1 [6]. The serine/threonine kinase glycogen synthase kinase 3 (GSK3) is another multifunctional target of AKT. Through phosphorylation, active GSK3 inhibits most of its substrates. Upon phosphorylation by AKT, GSK3 itself is inhibited and thereby the downstream targets are positively regulated. These targets include the prosurvival BCL-2 family member MCL-1 and the transcription factor c-Myc, which is required for expression of many genes involved in proliferation. Other GSK3-targets, such as glycogen synthase, are involved in (regulation of) cellular metabolism [7]. The third multifunctional target of AKT are the forkhead box O (FoxO) transcription factors. Phosphorylation of FoxO transcription factors by AKT leads to acute translocation out of the nucleus. Thus, AKT signalling suppresses the expression of FoxO targets. These include targets involved in the induction of apoptosis, cell-cycle arrest, catabolic metabolism and growth inhibition. Thus, PI3K-AKT signalling via these multifunctional targets promotes cell survival, growth and proliferation and steers cellular metabolism towards anabolism.

The PI3K-AKT pathway is regulated in many ways. The RAF-MEK-ERK pathway also promotes cell survival and growth and the two pathways have co-regulated proteins and negatively regulate each other. For example, MEK promotes membrane localisation of the phosphatase and tensin homologue (PTEN), where it dephosphorylates PIP3 and inhibits AKT activation [6]. Also, post-translational modifications (including (de)phosphorylation and acetylation) of AKT play important roles in regulation. An important immediate negative feedback loop is provided by mTORC1. Through a variety of downstream targets of mTORC1, AKT signalling is inhibited [7].

### 1.2. Alphaviruses

Alphaviruses, belonging to the family *Togaviridae*, are enveloped, icosahedral, positive-sense single-stranded RNA viruses. They are arthropod-borne, typically establishing persistent, asymptomatic infections in their vectors, which are usually mosquitoes, and acute infections in a broad range of vertebrate hosts, including humans, primates, horses and birds. They are globally distributed and can be distinguished into Old World and New World alphaviruses by geographical distribution and by clinical symptoms. Much of what is known of the biology of the Old World alphaviruses stems from decades of research on model viruses Semliki Forest (SFV) and Sindbis viruses (SINV), but the group also contains important human pathogens such as chikungunya virus (CHIKV) and Ross River virus (RRV). Currently, no vaccines or antiviral therapies have been approved for any alphavirus.

The viral genome is a single strand of positive-sense RNA with a 5′ 7-methyl-GpppA cap and a 3′ poly(A) tail. It contains two open reading frames (ORFs). The first ORF encodes the non-structural polyprotein (nsP) P1234 and is translated immediately after infection and the second, encoding the structural proteins, is translated later and to very high levels. All nsPs fulfil a role in viral RNA synthesis [8]. Those roles are quite well understood although the function of nsP3 remained obscure for a long time but is starting to be more understood [9,10]. nsP3 has three domains: the macrodomain, the alphavirus unique or zinc-binding domain (AUD/ZBD) and the hypervariable region (HVD). The macrodomain and AUD/ZBD are conserved among alphaviruses, while the HVD shows high variability in sequence and length even between closely related alphaviruses. The nsP3 HVD is a hub for interactions with multiple cellular proteins and can modulate multiple cellular pathways during infection [11].

### 1.3. Effect of Alphaviruses on PI3K-AKT Pathway During Infection

Several alphaviruses influence the PI3K-AKT(-mTOR) pathway during infection. For four of the Old World alphaviruses (SFV, RRV, CHIKV and SINV) PI3K-AKT activation has been studied and will be discussed in the following sections. Briefly, activation appears to manifest itself as either extremely strong or weak, depending on the virus. The complexity of PI3K-AKT pathway activation, cross-talk with other pathways and the numerous effectors, make it very difficult to appreciate what outcomes are important for replication of the different members of the alphavirus genus.

### 1.4. SFV and RRV Hyperactivate the PI3K-AKT-mTOR Pathway and Downstream Effectors

**Figure 2:**
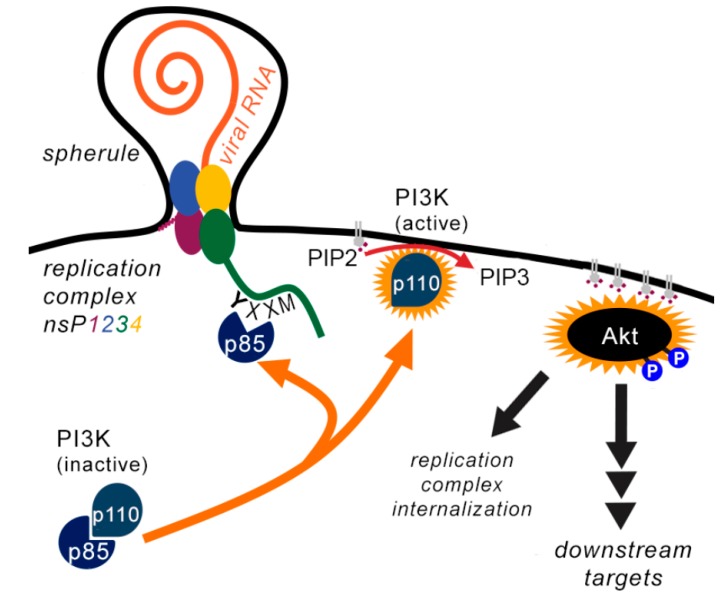
In Semliki Forest virus (SFV) and Ross River virus (RRV)-infected cells, viral replication complexes form at the PM. Via recruitment of p85 to a YXXM motif in nsP3, p110 is freed from regulation and can catalyse the conversion of PIP2 to PIP3 and recruitment and activation of AKT to the PM. Via multiple downstream effector pathways, the internalization of viral replication complexes as well as a heightened cellular metabolic activity and likely other yet undescribed states are promoted. For more details, see text.

Both SFV and RRV have been shown to very strongly activate the PI3K-AKT-mTOR pathway, via the presence of YXXM motifs in the C-terminal HVD of nsP3 (Y369-E370-P371-M372 for SFV, and Y356-E357-T358-M359 for RRV) [12] (Figure 2). YXXM motifs are known activators of class 1A PI3Ks [13,14] and, after localisation to the PM in nascent viral RNA replication complexes, the YXXM motifs of SFV and RRV nsP3 bind to one or both of the SH2 domains of p85, thereby releasing the enzymatic p110 subunit, leading to activation of AKT [12]. Strikingly, the hyperactivation of the pathway even occurs when cells are starved of amino acids and growth factors prior to infection. The presence of PM-associated nsP3 is sufficient to induce AKT phosphorylation [15], suggesting interaction with a PM-localised cellular factor important for this activation. By comparison with other YXXM-containing proteins, we propose that the tyrosine residue is phosphorylated by a PM-localised cellular tyrosine kinase, but the identity of such a kinase(s) is not known.

All alphaviruses encode nsP3, but the HVD greatly varies in length and sequence between alphaviruses. We analysed the nsP3 sequence of all alphaviruses and found that YXXM motifs are only present in SFV, RRV and the equine alphaviruses Getah virus and Sagiyama virus [12]. There are no studies showing whether the latter viruses also hyperactivate AKT in their respective host cells. Interestingly, some viruses from other families also activate AKT via YXX(X)M motifs, including influenza virus [16] and herpes simplex virus type 1 [17].

SFV infection leads to the induction of strong phosphorylation of AKT and two downstream targets of mTORC1, S6 and 4E-BP1 [15]. Furthermore, it induces phosphorylation of the AKT substrates phosphofructokinase 2 (PFK2), Rab GTPase-activating protein AS160 (or TBC1D4) and ATP citrate lyase (ACL) [12]. These latter substrates all play a role in cell metabolism. Treatment of infected cells with wortmannin, a specific PI3K inhibitor, reduces but does not eliminate phosphorylation of AKT, PFK2, AS160 and ACL [12,15]. This suggests that SFV-induced AKT hyperactivation is mediated by PI3K-dependent and independent mechanisms. S6 phosphorylation is not reduced upon wortmannin treatment, probably due to the fact that mTORC1 is also stimulated via other pathways [15].

For both SFV and RRV, wortmannin treatment inhibits viral replication at late stages only, suggesting that PI3K-AKT activation and consequent changes in cellular metabolism are more important for later stages of viral replication. For RRV however, there is no difference in release of new virions when cells are infected with an RRV mutant that cannot hyperactivate AKT. However, in a murine model of RRV infection, wildtype RRV causes a more severe disease than the non-hyperactivating mutant [12]. Probably, AKT activation is not essential for in vitro replication, but does contribute to infection in vivo. Effects of the pathway on SFV pathogenesis in vivo have not been studied.

### 1.5. CHIKV Activates PI3K-AKT Moderately

CHIKV infection of a number of different cell types and species also leads to activation of PI3K-AKT [18,19,20,21], and PI3K expression was found to be upregulated in total white blood cells isolated from CHIKV infected patients with both mild and severe disease [22]. As the PI3K inhibitor wortmannin completely blocks CHIKV-induced activation of AKT [15], the activation is likely mediated entirely via PI3K. However, when directly compared to SFV, the PI3K-AKT activation by CHIKV is significantly slower and less extensive [15]. In line with that, CHIKV nsP3 does not have a YXXM motif and likely does not hyperactivate by binding to SH2 domains of p85. The mechanism of this moderate activation of PI3K-AKT is not known. Src family kinases (SFK), which have been shown to be important for CHIKV-replication and with which CHIKV might interact [19], directly interact with AKT and are required for its activation [23,24]. It has been shown that microRNAs that target proteins in the PI3K-AKT pathway are modulated by CHIKV [21] and may play some role in the activation. Nevertheless, despite less extensive activation than SFV or RRV, the pathway appears to be important for replication and pathogenesis of CHIKV. Inhibition of PI3K has been shown to inhibit CHIKV replication, translation of CHIKV proteins and the amount of new infectious viruses [19,25].

In contrast, another study showed that inhibition of PI3K did not affect CHIKV replication, whereas direct inhibition of AKT or inhibition of protein kinase A (PKA), a kinase which regulates AKT-activation independent of PI3K, did inhibit CHIKV replication [18]. This might suggest that CHIKV could activate AKT independently of PI3K, through PKA. Another AKT inhibitor, GSK 690693, did not influence CHIKV replication [19]. CHIKV infection transiently inhibits activation of mTORC1 by increasing reactive oxygen species (ROS) levels [25,26]. Inhibition of mTORC1 with either rapamycin, TORISEL or RAD001 is reported to not affect CHIKV replication [18], and might even favour it [19,25]. Treatment of CHIKV infected human fibroblasts with dasitinib strongly reduced viral titres by blocking translation of subgenomic RNA [19]. This might be through PI3K-AKT activation, but also through MAPK activation, as MAPK signalling pathways were shown to be required for optimal replication of CHIKV [27]. However, inhibition of both mTORC1 and mTORC2 by Torin 1 decreases CHIKV replication, suggesting that CHIKV needs mTORC2, though it does not depend on mTORC1 [19]. mTORC2 activates AKT, but also other kinases, such as protein kinase C isotypes and serum and glucocorticoid-induced kinase 1 (GSK1) [28]. SFK and mTORC1/mTORC2 seem to be important for more alphaviruses, as Mayaro virus (MAYV), o’nyong’nyong virus (ONNV) and RRV titres are also decreased upon inhibition with dasitinib or Torin 1 [19]. The contradicting results of different inhibitors on CHIKV replication might be due to the relatively weak activation of the PI3K-AKT pathway, which may be more sensitive to variation.

### 1.6. SINV Differentially Activates PI3K-AKT in Different Species

Lacking a YXXM motif, SINV also does not hyperactivate the PI3K-AKT pathway [12], but rather appears to induce low or transient activation in cell lines from different species [12,29,30]. LY294002, which inhibits class I PI3Ks [31], inhibits SINV RNA replication, but not virus titres in human cells [30]. Specific mTORC1 inhibition does not decrease SINV replication [30], and might even increase it [25]. Taken together, these reports do not suggest a strong requirement for the PI3K-AKT-mTOR activation for efficient SINV infection and replication.

### 1.7. Benefits of Activation of PI3K Pathway for Alphaviruses

As described above, activation of the PI3K-AKT pathway can have many effects on diverse cellular processes, including cell survival, growth and proliferation and metabolism, and many more functions specific for certain cell types [7]. In the subsequent sections, we discuss what is known about the effects of alphavirus-induced PI3K-AKT activation on vertebrate cells.

## 2. Metabolic Change

### Alphaviruses Influence Cellular Metabolism

PI3K-AKT activation by SFV leads to a change in cellular metabolism, probably through increased phosphorylation of AKT targets. It increases glycolysis and causes an increased glucose flux through the tricarboxylic acid cycle (TCA). Consequently, more citrate is exported to the cytoplasm and more fatty acids are de novo synthesised. Furthermore, there is increased activation of the pentose phosphate pathway (PPP), which results in increased synthesis of nucleotides, such as UMP [12]. Treating cells for 16h with the glucose analogue 2-deoxyglucose (2-DG), which inhibits glycolysis and prevents entrance of glucose into the PPP, followed by SFV infection, strongly reduces production of new virions [12,32]. This effect is still seen when 2-DG is administered at 3 hpi, which indicates that glycolysis is not required for early events in replication [32]. Inhibition of the PPP specifically through the glucose-6-phosphate dehydrogenase inhibitor (DHEA), reduces production of new virions almost 10-fold. Furthermore, treatment of infected cells with wortmannin or the AKT-inhibitor MK-2206 dramatically inhibits glycolysis and reduces the release of new virions. This indicates the importance of the metabolic shift for SFV replication [12].

RRV infection increases glycolysis moderately in a manner that appears to be independent of the hyperactivation of PI3K-AKT [12]. In contrast, wildtype RRV increases fatty acid levels significantly, whereas RRV-YF (which does not hyperactivate PI3K-AKT) does not. It is not shown whether inhibition of fatty acid synthesis hampers RRV replication. RRV-YF is attenuated in an in vivo mouse model, but this attenuation is not directly attributed to effects on metabolism [12].

Conversely, although SINV does not induce sustained AKT phosphorylation in human cells, it does activate glycolysis at 8 hpi [12]. SINV also increases glycolytic flux in mouse neuroblastoma cells (Neuro 2a), perhaps to compensate for virus-induced mitochondrial dysfunction [33]. Inhibition of glycolysis or PPP in SINV-infected cells strongly decreases the release of progeny virus in both human and African green monkey kidney (Vero) cells [12,32]. Similar to SFV, glycolysis is not required for virus entry, but is important later during the viral life cycle [32]. It does not seem that SINV infection increases fatty acid synthesis. Thus, SINV induces a slightly different metabolic profile, but needs glycolysis and PPP for replication in cells of various species. SINV does not induce sustained AKT-activation in human cells, and probably has evolved other mechanisms to stimulate those metabolic pathways [12].

Metabolic pathways have not been well studied in the context of CHIKV infection. There are some indications that CHIKV activates glycolysis. PKM2, an isoenzyme of the glycolytic enzyme pyruvate kinase, is overexpressed in CHIKV-infected mouse muscles [34]. Furthermore, in human hepatic cells, CHIKV induces upregulation of PDHA1, a subunit of the pyruvate dehydrogenase complex involved in transforming pyruvate to acetyl-CoA in the TCA. However, other glycolytic and TCA-enzymes, such as alpha-enolase and isocitrate dehydrogenase, are downregulated in CHIKV-infected cells [35]. Regarding the other alphaviruses, only MAYV is known to increase glycolytic flux strongly, through activation of the enzyme 6-phosphofructo 1-kinase (PFK1) [36]. However, it is not shown whether MAYV activates AKT.

To conclude, all abovementioned alphaviruses influence cellular metabolism. However, this is not always entirely AKT-dependent, and all alphaviruses modulate cellular metabolism slightly differently. Processes such as increased glycolysis are essential for some viruses, but dispensable for others, at least in vitro. Further study will be necessary to gain a more complete understanding of the importance of metabolic alterations to alphavirus infections.

Many viruses influence cellular metabolism in order to create optimal circumstances for replication. Most viruses stimulate glycolysis and the PPP and fatty acid synthesis. Through these mechanisms, viruses can increase the availability of energy and building blocks for replication and progeny production [37]. During acute infection, viruses need much energy and building blocks for viral replication and production of viruses. Thus, it is very plausible that alphaviruses as well as other viruses causing acute infection, activate PI3K-AKT to influence cellular mechanism and ensure this. However, PI3K-AKT-dependent increased glucose uptake and glycolysis was also shown to promote an interferon-induced antiviral response [38]. Increased glycolysis is beneficial for the virus but comes at a cost.

## 3. Interaction with Autophagy

### 3.1. Various Viruses Influence Autophagy

Macroautophagy, the process by which intracellular components, such as long-lived proteins and organelles are degraded and recycled is also regulated at least in part by the PI3K-AKT pathway [39]. It is a constitutive process but can be further induced through activation of the kinase Ulk1 by AMP activated protein kinase (AMPK). Activation of the PI3K-AKT-mTORC1 pathway inhibits autophagy through inhibition of the interaction between Ulk1 and AMPK by mTORC1 [40].

Autophagy in the context of virus infection has been well studied, although it is not always clear if the effects of the pathway are pro- or antiviral [41,42]. The benefits of autophagy seem to be different for different viruses, and dependent on the time after infection. Some viruses, such as ZIKV and rotavirus require the process early in infection [43,44,45,46], whereas this is detrimental for others. Conversely, some viruses, such as influenza A virus induce autophagy late after infection to increase replication [47]. Finally, there are viruses in which autophagy does not appear to influence replication. Autophagy has been studied in the context of CHIKV, SFV and SINV infection, though most of these studies did not investigate the direct role of PI3K-AKT activation. These alphaviruses appear to have different effects on autophagy, though there also might be variation due to different cell types used and different experimental conditions.

### 3.2. CHIKV Induce Autophagy, Whereas SFV Blocks It

CHIKV infection increases the amount of autophagosomes in various human cell lines: human embryonic kidney (HEK) 293 cells [48], HeLa cells [49,50], human glioblastoma cells (U-87) [51], and murine embryonic fibroblasts (MEF) [26] from early in infection and continuously increasing during the course of infection. CHIKV induces oxidative stress, leading to increased intracellular ROS and NO levels. This inhibits mTORC1, which results in increased autophagy [26]. CHIKV-induced autophagy has the effect to reduce or delay CHIKV-induced cell death [26]. The correlation between increased autophagy and efficient CHIKV replication might be species dependent [26,51,52]. An explanation for the difference between species came with the discovery that in human cells, CHIKV nsP2 interacts with the autophagy receptor NDP52, but not in murine cells [50]. The situation appears more complicated in vivo, where one study reported that induction of autophagy attenuates disease, when compared between wildtype and autophagy-deficient mice [26], while another showed that inhibition of autophagy with Tat-beclin 1 peptide reduces viral titres and improves clinical outcomes in CHIKV-infected mice [52].

SFV also increases the amount of autophagosomes in the infected cell [53]. However, in contrast to CHIKV, SFV infection does not appear to strongly affect the production of autophagosomes but rather blocks their degradation in a manner dependent on expression of the viral glycoproteins. Since SFV replication was unaffected by ATG5 depletion, it is concluded that the pathway plays no role in replication in vitro [53].

In conclusion, many acute viruses influence AKT and mTORC1 activation to influence autophagy. For most alphaviruses it is not clear how PI3K-AKT activation relates to autophagy. SFV strongly activates PI3K-AKT-mTORC1, but specifically blocks autophagosome degradation, which seems contradictory as active mTORC1 blocks both autophagosome formation and degradation. CHIKV activates PI3K-AKT moderately, but inhibits mTORC1 via ROS and NO, resulting in increased autophagy.

## 4. Promotion of Cell Survival

### Unknown Whether Alphaviruses Activate PI3K-AKT to Promote Cell Survival

Many AKT substrates play a role in promoting cell survival and proliferation, but there are currently no studies addressing the effects of AKT on cell death in alphavirus infection. The New World alphavirus Venezuelan equine encephalitis virus stimulates early growth factor response genes in infected astrocytes, but this was shown not to be dependent on MAPK or PI3K signaling [54]. Rubella virus (RV) which also belongs to the *Togaviridae* family, induces caspase-dependent apoptosis, leading to a cytopathic effect, but delays and reduces this process through PI3K-AKT activation [55]. However, in our preliminary experiments, we did not detect any significant delay in cell death in wildtype SFV infection compared to mutants, which do not hyperactivate the pathway (unpublished).

## 5. The Strange Case of the Trafficking of Replication Complexes

### Some Alphaviruses Stimulate Internalisation of Replication Complexes

Early in infection, alphavirus replication complexes (RC) assemble in membrane invaginations/spherules at the PM in which viral replication takes place [56,57]. A striking effect of PI3K-AKT hyperactivation by SFV and RRV is the trafficking of RC from the PM. First, RC localise in small and scattered cytoplasmic vesicles and then in large acidic perinuclear vacuoles (called cytopathic vacuoles of type I (CPV-I)). In SFV infection, RCs are relocalised from the PM to CPV-I at 8 hpi [56]. When PI3K is inhibited or when the YXXM motif in nsP3 is mutated, trafficking of RC does not take place [12,15,56]. It is not known which downstream targets of AKT mediate RC trafficking. Remarkably, viral RNA synthesis is not hindered when PI3K is inhibited, indicating that RC at the PM alone can sustain RNA replication [15]. Therefore, it is difficult to determine whether internalisation of RCs has benefits for the virus.

In other alphaviruses, the effect is less clear. CHIKV activates PI3K-AKT, although to a lesser extent than SFV, and CHIKV RCs mostly remain at the PM [15]. RC of SINV, which does not activate PI3K-AKT in human cells, but does activate it in murine cells, partially relocalises into the cytoplasm in baby hamster kidney cells (BHK-21), though the majority of RCs remain PM membrane associated [57]. Altogether, it seems that only strong AKT activation is required to induce trafficking of alphavirus RCs and replication happens to a similar extent in RC at the PM as in CPV-I.

## 6. Remarks in Conclusion

Alphaviruses are pathogens of growing importance, causing increasing numbers of cases throughout the world but for which there are no vaccines or antiviral therapies. We have reviewed the literature showing that several alphaviruses activate the PI3K-AKT pathway in vertebrate cells. The extent of activation differs (Table 1), as SFV and RRV induce hyperactivation and CHIKV causes moderate activation. SINV does not induce sustained AKT activation in humans but activates AKT in mice. The mechanism of hyperactivaton by SFV and RRV is known [12], and future work will reveal the mechanism of the moderate activation by CHIKV, SINV and mutants of SFV and RRV which do not hyperactivate. Inhibition of PI3K-AKT most clearly inhibits replication of SFV and RRV, while for CHIKV there are contradicting results and SINV does not seem to rely on PI3K-AKT for replication.

Thus, PI3K-AKT activation has several (potential) benefits for alphaviruses, of which changing the cellular metabolism seems to be the most important. However, PI3K-AKT-mTORC1-S6K mediates an interferon-induced antiviral response [58]. Furthermore, AKT contributes to the activation of NF-κB, which leads to production of inflammatory cytokines [2]. This is not studied in alphavirus-infected cells, but it is very important to determine to which extent PI3K-AKT is needed for antiviral immunity and when PI3K-AKT activation is beneficial for the virus, but detrimental for the host. In development of antiviral therapies, proteins from the PI3K-AKT pathway might be good targets. However, too strong inhibition of PI3K-AKT will probably damage the host, as cells will become much more prone to apoptosis and innate immune pathways may be induced. Therefore, targeting proteins that play a role in the processes that AKT stimulates and are beneficial for alphaviruses, such as anabolic metabolism, is also an important subject for further research.

## Figures and Tables

**Table 1:** Mechanisms and Effects of PI3K-AKT activation.

	Mechanism of PI3K-AKT Activation	Effects of PI3K-AKT Activation on			
		Metabolism	Autophagy	Apoptosis	Trafficking RC
**SFV**	Strong activation via YXXM motif in nsP3	Increases glycolysis and fatty acid synthesis	Blocks degradation of autophagosomes	Small, not significant delay	RCs traffic from PM to CPV-I
**RRV**	Strong activation via YXXM motif in nsP3	Increases fatty acid synthesis	Unknown	Small, not significant delay	RCs traffic from PM to CPV-I
**CHIKV**	Moderate activation by unknown mechanism	Unknown	Increases production of autophagosomes	Unknown	RC mostly remain at PM
**SINV**	Weak or transient activation by unknown mechanism	Unknown	Unknown	Unknown	RC mostly remain at PM
